# Comparison of different Pancreatic cancer treatments: a three-year retrospective study in the oncology center of Tangier university hospital, Morocco

**DOI:** 10.1186/s12876-023-03071-0

**Published:** 2023-12-21

**Authors:** Houda Abrini, Mounia Amzerin, Aicha El Baaboua, Sara Aboulaghras, Alia Bouhda, Fatima Zahra El Mrabet

**Affiliations:** 1 Department of Medical Oncology, Faculty of Medicine and Pharmacy, Ahmad Bin Zayed Al Nahyan Center of Cancer Treatment, Mohammed VI University Hospital of Tangier, Abdelmalek Essaadi University, Tangier, Morocco; 2 Biotechnology and Applied Microbiology Team, Department of Biology, Faculty of Sciences, Abdelmalek-Essaadi University, Tetouan, Morocco; 3 Physiology and Physiopathology Team, Genomic of Human Pathologies Research, Faculty of Sciences, Mohammed V University, Rabat, Morocco

**Keywords:** Pancreatic cancer, Chemotherapy, Folfirinox, Gemcitabine, Retrospective study, Morocco

## Abstract

**Background:**

Pancreatic cancer is among the most lethal malignancies, with a 5-year overall survival (OS) of less than 10% for all stages. The present study aims to evaluate the epidemiological and clinical characteristics, as well as the results of different treatments of patients diagnosed and treated between 2019 and 2021 in the Oncology Center of Tangier, University Hospital, Morocco.

**Methods:**

To compare the evolution of the pancreatic cancer between the different chemotherapy regimens, a retrospective study was performed using data collected over a period of 3 years. For each patient, the data were described and statistically analyzed in the dedicated operating sheet.

**Results:**

55 pancreatic cancer patients were included in this study, and the median follow up was 3 months. The mean age of patients was 59.5 ± 10.3 years (extremes 34–79) and the sex ratio male/female was 0.9. Most patients were diagnosed with adenocarcinoma (92.3%), but metastatic stage was the most frequent (56.4%). The surgery was applied to 16.36% of patients. 10.9% of patients have received adjuvant chemotherapy and 76.4% received palliative chemotherapy. Chemotherapy regimens included mainly Gemcitabine and Folfirinox. The median OS was significantly longer for patients treated with Folfirinox versus Gemcitabine (6 months versus 3 months, *p-value* < 0.016). The median OS for patients that received Folfirinox and Gemcitabine successively (19.7 months) was significantly longer compared to patients that received a monotherapy with either Folfirinox or Gemcitabine alone (*p-value* < 0.016).

**Conclusion:**

These findings reinforce the use of advanced methods for earlier detection of pancreatic cancer and the development of effective immunotherapies or more targeted therapies.

## Background

Pancreatic cancer is highly lethal, with the mortality to incidence ratio is 90% [1]. It is the 7th most common cause of cancer-related death and has a very poor prognosis, with the 5-year overall survival rate is only 10% [2]. The difficulty of prevention is due to the lack of specific risk factors and late diagnosis because of the absence or non-specificity of early clinical signs or reliable biological markers, as well as the speed of tumor spreading [3]. Surgery is the only hope for a cure, but it is curative in only 15–20% of cases [4].

The incidence and mortality rates of pancreatic cancer are high in countries with high Human Development Index, and low in the ones with low and middle-incomes [5]. African and South-Central Asian countries have the lowest age-standardized rates [6]. Pancreatic cancer is usually more prevalent in men than women, and its incidence and death rates increase with age [7].

Adenocarcinoma is the most common histological type of pancreatic cancer [8]. Obviously, most therapeutic approaches employed become useless once the disease progresses to an incurable level [9]. That is why, widespread demand for early detection, quick diagnosis and treatment of the tumor have been emphasized by many health care organizations [10]. Unfortunately, the great majority of pancreatic cancer malignancies are diagnosed in metastatic phase.

In the present retrospective study, we analyzed the epidemiological, clinical, and therapeutic characteristics and the prognosis of pancreatic cancer, as well as the therapeutic regimens deployed in the Oncology Center of Tangier University Hospital, Morocco. To the best of our knowledge, this is the first study carried out in the North of Morocco about pancreatic cancer.

## Methods

### Study interest

The present study was designed first to report the epidemiological and clinical characteristics, and then to compare the efficacy of the different treatments used in patients with pancreatic cancer at Oncology Center of the University Hospital of Tangier, northern Morocco.

### Patients

This retrospective study included all patients diagnosed and treated for pancreatic cancer in above-mentioned center between April 2019 and November 2021. No pancreatic cancer patient was excluded from the study. The medical records of patients were reviewed, and data files were extracted and collected in Microsoft Excel spreadsheets (Microsoft Corp. Redmond, WA, USA, 2013). This study was approved by Tangier University Hospital Ethics Committee (AC31JL/2023). The patients included in this study were treated by oncologists using chemotherapy regimen of Folfirinox and Gemcitabin according to ESMO and NCCN guidelines.

### Data collection

Personal profile of each patient, including frequency of visits, gender, age and history were collected. Clinical reports including consultation periods, functional signs and clinical examination, and para-clinical tests such as histological, radiological and biological examination were gathered using dedicated operating sheet. In addition, the descriptions of the different therapeutic approaches were included in the present study: regimens of chemotherapy in adjuvant and metastatic stage, surgery and endoscopy, as well as follow-up period, progression time and survival time.

### Statistical analysis

The collected data were analyzed using IBM SPSS Statistics 21 software (SPSS Inc., Chicago, IL, USA). Means and standard deviation as well as the percentages of patients of each age, socio-demographic information, clinical and histological studies, therapeutic aspects were calculated and described in this work. Median overall survivals (OS) were calculated using Kaplan-Meier method. *P-value* was calculated using the no stratified Log-Rank test when comparing OS between every two groups of patients, e.g., the Gemcitabine versus the Folfirinox group and for the dual therapies with Gemcitabine and Folfirinox versus Gemcitabine or Folfirinox. *P-value <* 0.05 was considered statistically significant.

## Results

### Socio-demographic data

During a period of 3 years, 55 patients, with 26 male and 29 female (sex ratio: 0.9), were treated for pancreatic cancer in the Oncology Center -Tangier University Hospital, Morocco.

The average number of pancreatic cancer patients recruited per year was 18.3 patients (Table 1). The mean age was 59.5 ± 10.3 years with extremes ranging from 34 to 79 years. 43% of patients were 65 years or older. Moreover, 9% of patients were under 44 years of age. The analysis of patients’ files showed that 14.5% of patients were under treatment of diabetes and the other 12.7% of patients were chronic smokers. None of the patients were alcohol consumer and none of the patients had family history of pancreatic cancer (Table 1).

**Table 1:** Demographic data for pancreatic cancer patients

Variable	Cancer patients (n = 55)	
	N_o_	%
**Cases across the 3 years:**		
**2019**	15	27.3
**2020**	21	38.2
**2021**	19	43.6
**Average per year**	18.3	
**Male**	26	47.3
**Female**	29	52.7
**Age**:		
**Median (SD)** **Range**	59.5 *±* 10.3 34–79	
**Diabetes**	8	14.5
**Smokers**	7	12.7
**Alcohol consumption**	0	0
**Family history of pancreatic cancer**	0	0

N_0_ = Number of patients; SD: Standard deviation

### Clinical characteristics

The most frequent pancreatic cancer signs reported were epigastralgia, cholestatic jaundice and altered general condition (80% of patients). Furthermore, it was observed that 70% of the patients exhibited a performance status (PS) ≤ 2, of which 11% had PS 2, 16.4% experienced diffuse abdominal pain, 18.2% reported epigastric pain, and 11% presented right flank pains. Mucocutaneous jaundice was also noted in 11% of the patients, within the same group.

The most common histological type was adenocarcinoma (92.3%) (Table 2).

**Table 2:** Clinical features of the surveyed pancreatic cancer patients

Cancer patients (n = 55)				
Clinical features		Findings	No	%
**Features**	**Biopsy**	Adenocarcinoma	49	92.3
	**Surgery**		9 18 30	16.4 33.7 55.5
	**Endoscopy**			
	**Ca19-9 positive**			
	**Cancer seats CT TAP**	Cephalic tumor Corporeal cancer Caudal tumor	26 21 10	48 38 18
	**Cancer staging**	Metastatic stage Locally advanced Localized	31 15 9	56.4 27.3 16.4
**Chemotherapy**	**Adjuvant Chemotherapy**:		6	10.9
		Gemcitabine Folfirinox	5 1	83.3 16.7
	**Palliative Chemotherapy**:		42	76.4
		Gemcitabine Folfirinox	20 15	47.6 35.7

The results of radiological exam in pancreatic cancer patients varied depending on the type of imaging used and the stage of the cancer in these patients. From the 55 patients’ files analyzed, 90% had computed tomography thoraco-abdominal-pelvic (CT TAP), and the investigation of radiographic images revealed the location of the tumors at the head of the pancreas for 48% of patients, at the body for 34%, and at the tail of the pancreas for 18% of patients. Only 20% of patients performed hepatic magnetic resonance imaging (MRI). 56.4% of patients were in metastatic stage, 27.3% of patients had locally advanced cancer, and 16.4% had localized tumors (Table 2). The blood tumor marker Ca19-9 was positive in 55% of patients.

### Treatments

In the current study, 16.4% (n = 9) of patients had undergone a surgery of duodenopancreatectomy - cephalic with lymph node dissection. 33.7% (n = 18) of patients had biliary prosthesis by endoscopy.

**Fig. 1:**
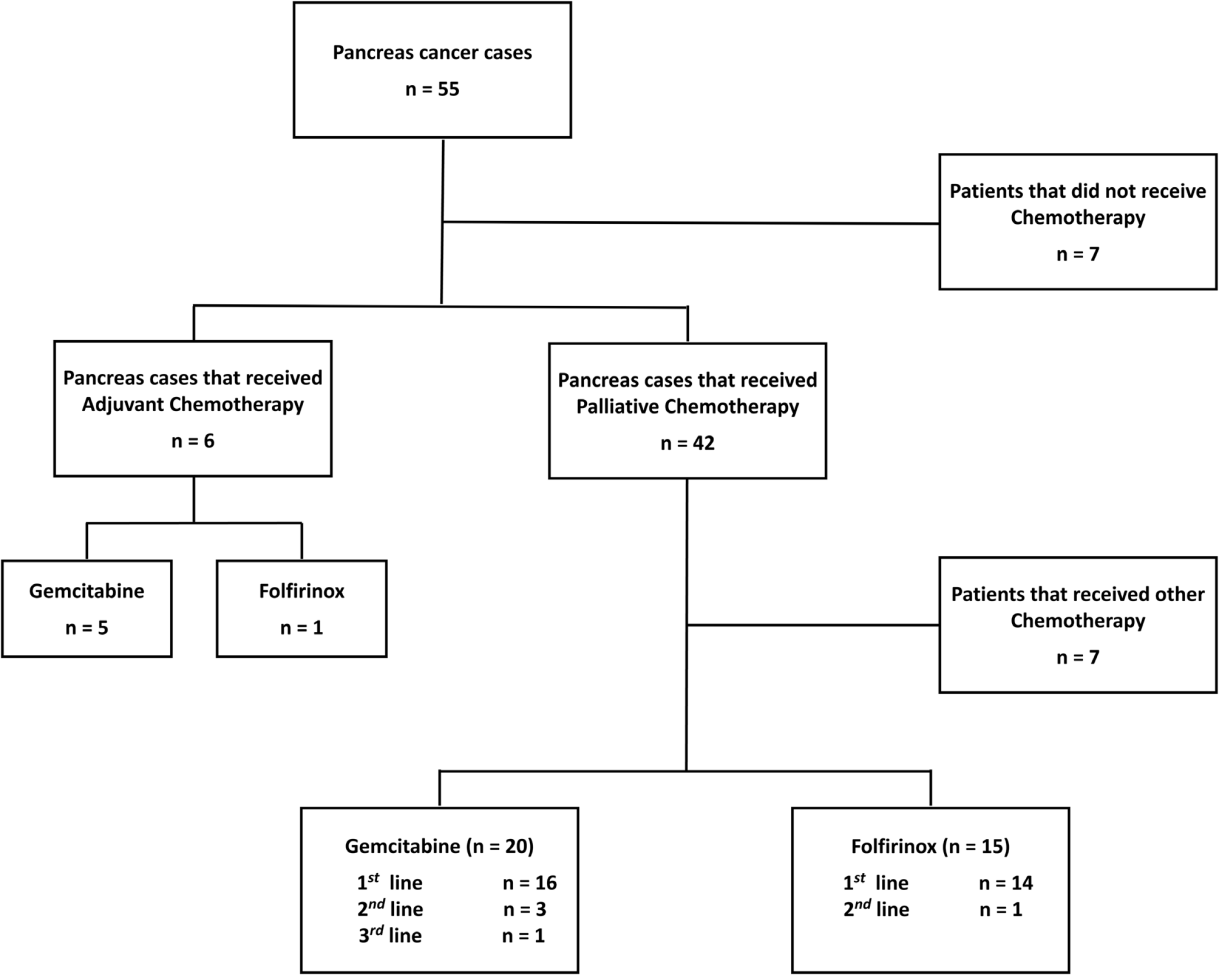
Chemotherapy regimens used for the treatment of pancreatic cancer in the Center of Oncology, Tangier University Hospital, Morocco

Regarding chemotherapy (Table 2; Fig. 1), Gemcitabine was the most used regimen. 10.9% of patients (n = 6) received adjuvant chemotherapy, 83.3% (n = 5) received Gemcitabine and 16.7% of patients (n = 1) received Folfirinox. 76.4% of patients (n = 42) received palliative chemotherapy, where Gemcitabine was administered in 47.6% of patient (n = 20) and Folfirinox was employed for 36% of patients (n = 15). The remaining seven patients (16.7%) received other chemotherapy regimens. For the 20 patients who received Gemcitabine, 16 received it in first line, three in second line and one in third line after progression. For patients in the Folfirinox group, 14 patients received it as first-line and one patient received it as second-line. Other patients of this cohort (n = 7) did not receive chemotherapy treatment because their PS did not allow it. The median follow-up time was 3 months.

### Survival

**Fig. 2:**
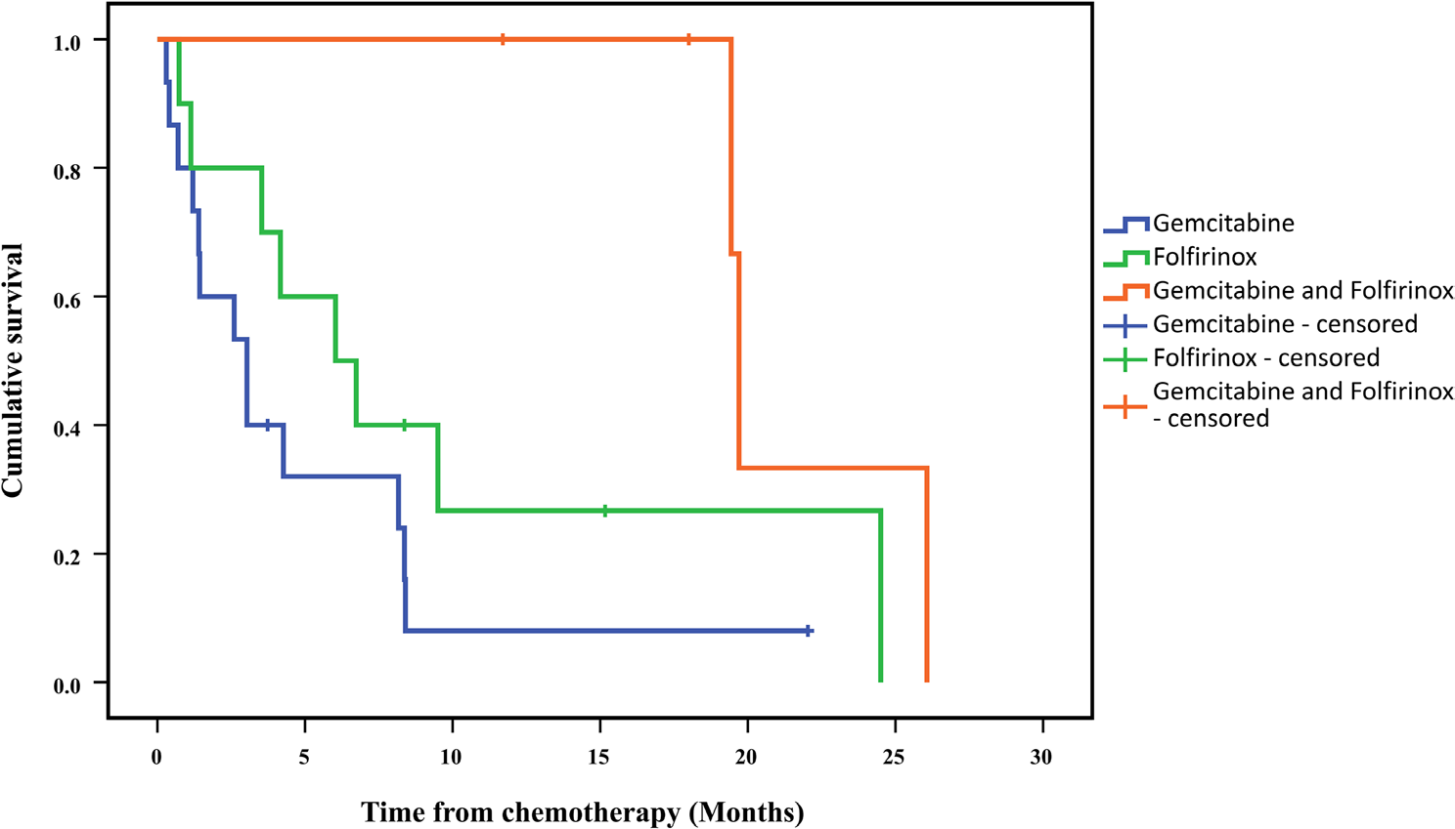
Kaplan-Meier curves for overall survival estimates in the Folfirinox, Gemcitabine and Folfirinox-Gemcitabine in relay

The Kaplan-Meier curves of Gemcitabine and Folfirinox groups (Fig. 2) fell very quickly and hence the death occurred very quickly as well.

These data revealed the severity of this disease. The median overall survival (OS) in the group of patients treated with Folfirinox was 6 months and in Gemcitabine group it was 3 months. The median OS in Folfirinox group was significantly longer than in Gemcitabine group with a *p-value* < 0.016. Likewise, the median OS in the group treated with both Folfirinox and Gemcitabine (19.7 month) was significantly longer than the groups treated with Folfirinox or Gemcitabine monotherapies ( *p-value* < 0.016, Table 3).

**Table 3:** Median overall survival (OS) of pancreatic cancer patients after each treatment

Types of treatment	Folfirinox	Gemcitabine	Gemcitabine-Folfirinox	*p-value*
**Median OS (months ± SD)**	6.03 ± 2.02	3.03 ± 1.01	19.70 ± 0.22	< 0.016*

SD: Standard deviation

*The *p-value* was smaller than 0.016 for the three types of treatment

## Discussion

In the present study, the average age of patients was 59.5 years. However, the reported average age in most studies was much higher. For example, the average age reported by Ramia-Angel et al. (2022) was 69.2 years [11]. The young age group (under 44 years) was a minority, accounting for less than 10%, as reported by Primavesi et al., (2019) [12]. The data explored in this study showed that the frequency of females affected by pancreatic cancer was slightly higher than that of males. These data are broadly consistent with those of Ferlay et al. (2021) [13], who reported that the frequency of patients with pancreatic tumors is approximately the same between males and females. Although previous studies by researchers at the Military Hospital Moulay Ismail in Meknes, Morocco have shown that males are at higher risk of developing the disease than females [14].

In the present study, 14.5% of patients had a history of diabetes. A positive link was observed between diabetes and the risk of pancreatic adenocarcinoma [15].

12.7% of patients had a history of smoking. This smoking rate is similar to previously reported rates in the Western region of Morocco [16], but lower than that reported by Hadizadeh et al., (2014) [17] in Iran (21.4%) and Zheng et al., (2016) [18] in China (35%). Smoking is one of the main risk factors for developing this type of cancer, with smokers having about twice the risk of developing pancreatic cancer compared to non-smokers [17, 18]. This risk increases with the number of cigarettes smoked per day. In fact, toxic chemicals in cigarettes can reduce immunity and damage DNA, leading to tumor development in the pancreas [19].

In current investigation, 90% of the patients underwent a computed tomography thoraco-abdominal-pelvic (CT TAP), which revealed the location of the tumors in the pancreas. 48% of patients had the tumors located at the head of the pancreas, 34% at the body, and 18% of patients had tumors at the tail of the pancreas.

In this study, the most frequent histological type of pancreatic cancer was adenocarcinoma (92.3%), which is in line with the data from the literature. A small number of patients (7.7%) presented neuroendocrine or acinar cell carcinoma. Biopsy is crucial for the diagnosis and monitoring of pancreatic cancer progression [20]. It is a reliable practice in detecting pancreatic cancer at an early stage, as it can diagnose recent or suspected cancerous pancreatic cells and can determine if other organs are affected [20].

The metastatic stage was the most observed stage in this study (56.4%), indicating that pancreatic cancer was diagnosed in patients at an advanced stage due to its aggressive behavior, tumor biology, and frequency of nonspecific symptoms.

55% of patients were tested positive for CA19-9 which is a useful marker for monitoring the status of the disease before and after pancreatic resection [21]. An increase in the concentration of this tumor marker indicates the progression of the cancer stage, mainly the metastatic stage [21].

Pancreatic surgery was performed in nine patients, indicating that it was relatively uncommon, likely due to delayed diagnosis limiting therapeutic options. Surgery is the main realistic option for treating pancreatic cancer by removing malignant tumors to alleviate cancer-caused symptoms and improve patients’ quality of life [22]. 32% of patients had undergone endoprosthesis that has many advantages in terms of cost and comfort compared to surgical treatment [23]. Endoprosthesis is known to improve the quality of life of patients that suffer from pruritis, vomiting, nausea, and jaundice caused by bile duct blockage due to pancreatic tumors [24].

Adjuvant chemotherapy is the most recommended approach in early stages of pancreatic cancer. It can reduce cancer progression and the risk of recurrence after surgery. However, palliative chemotherapy is often used in advanced or terminal stages of cancer and aims to treat, relieve symptoms, and improve the quality of patients’ life. The two drugs used for the treatment of our patients were Gemcitabine and Folfirinox, both used in adjuvant and palliative chemotherapies. The median overall survival of patients treated with Folfirinox was significantly longer than that with Gemcitabine. This is in line with the results reported by Klaiber et al., [25] Who showed that adjuvant modified Folfirinox increased the 5-year survival rate by 50%. Likewise, in a multicenter phase III study (PRODIGE 4/ACCORD 11), the Folfirinox regimen showed a significant improvement in overall survival compared to Gemcitabine monotherapy (11.1 months versus 6.8 months) [26]. The use of Gemcitabine as first-line treatment increased median overall survival of patients (5.6 months) compared to those treated with fluorouracil (4.4 months), representing an 18% statistically significant improvement [27].

The efficiency of chemotherapy increased in the group that received Folfirinox followed by Gemcitabine. This is consistent with the literature. Indeed, Shi and Yu (2019) [28] evaluated the effectiveness of different chemotherapy regimens in patients with pancreatic cancer and established that chemotherapy regimens including the combination of 5-fluorouracil, irinotecan, and oxaliplatin were more effective treatment for patients compared to regimens including Gemcitabine alone. The administration of Folfirinox regimen supplemented with growth factors is a current standard treatment for patients with good performance status (0–1) [26]. Similarly, the combined treatment with nab-paclitaxel and Gemcitabine administered in the first line to patients with metastatic pancreatic adenocarcinoma showed significant improvements in median overall survival compared to Gemcitabine alone (8.5 months vs. 6.7 months) [29]. The combined adjuvant chemotherapy with Folfirinox, Gemcitabine and nab-paclitaxel proved to be effective in patients with recurrent pancreatic cancer [30]. In this retrospective study, the median overall survival (OS) was significantly longer for patients treated with Folfirinox (6 months) compared to Gemcitabine (3 months). Additionally, the median OS for patients that received Folfirinox and Gemcitabine successively was significantly longer (19.7 months) compared to patients that received a monotherapy with either Folfirinox or Gemcitabine.

## Conclusion

In the current retrospective study, the results have confirmed the poor prognosis of pancreatic cancer, as evidenced by the observed metastatic stage. Most patients received palliative chemotherapy rather than adjuvant chemotherapy, with Gemcitabine being the most administered regimen. The median OS of patients in the Folfirinox group was longer than that in Gemcitabine group in both adjuvant and first-line metastatic treatment. This improvement was also observed in patients that received Folfirinox and Gemcitabine successively. These results support the use of advanced methods for earlier diagnosis of pancreatic cancer to be able to offer surgery to a big number of patients, keep the patients in good general condition to benefit from optimal chemotherapy, and to develop advanced therapies.

## Data Availability

The datasets analyzed during the current study are available from the corresponding author on reasonable request.

## Abbreviations

Ca19-9: Cancer antigen 19 − 9
CT TAP: Computed tomography thoraco-abdominal-pelvic
DNA: Deoxyribonucleic Acid
MRI: Magnetic resonance imaging
OS: Overall survival
PS: Performance status
